# The Hippo pathway in disease and therapy: cancer and beyond

**DOI:** 10.1186/2001-1326-3-22

**Published:** 2014-07-10

**Authors:** Marta Gomez, Valenti Gomez, Alexander Hergovich

**Affiliations:** 1 Tumour Suppressor Signalling Networks laboratory, UCL Cancer Institute, University College London, 72 Huntley Street, WC1E 6BT London, UK

**Keywords:** Hippo signalling cascade, Protein kinases, Scaffolding proteins, Protein-protein interactions, Cancer, Therapy, Tissue repair, MST, LATS, MOB1

## Abstract

The Hippo tumour suppressor pathway co-ordinates cell proliferation, cell death and cell differentiation to regulate tissue growth control. In mammals, a conserved core Hippo signalling module receives signal inputs on different levels to ensure the proper regulation of YAP/TAZ activities as transcriptional co-activators. While the core module members MST1/2, Salvador, LATS1/2 and MOB1 have been attributed tumour suppressive functions, YAP/TAZ have been mainly described to have oncogenic roles, although some reports provided evidence supporting growth suppressive roles of YAP/TAZ in certain cancer settings. Intriguingly, mammalian Hippo signalling is also implicated in non-cancer diseases and plays a role in tissue regeneration following injury. Cumulatively, these findings indicate that the pharmacological inhibition or activation of the Hippo pathway could be desirable depending on the disease context. In this review, we first summarise the functions of the mammalian Hippo pathway in tumour formation, and then discuss non-cancer diseases involving Hippo signalling core components with a specific focus on our current understanding of the non-cancer roles of MST1/2 and YAP/TAZ. In addition, the pros and cons of possible pharmacological interventions with Hippo signalling will be reviewed, with particular emphasis on anti-cancer drug development and regenerative medicine.

## Introduction

In complex multicellular organisms, normal tissue development, repair and maintenance is essential for organ functionality and consequently the survival of the organism. To ensure that these complex biological events are performed accurately, cell proliferation, cell death and cell differentiation (and de-differentiation) must be coordinated by cellular signalling mechanisms. Research performed mainly over the past decade has uncovered that the Hippo tumour suppressor pathway is a master regulator of proliferation, death and differentiation [1]. Therefore, intensive research efforts have been invested to understand the molecular function and regulation of Hippo signalling. Although *Drosophila* genetics have been an instrumental driving force in obtaining our current level of knowledge of Hippo signalling [2], we will focus in this review only on the mammalian Hippo pathway.

**Figure 1:**
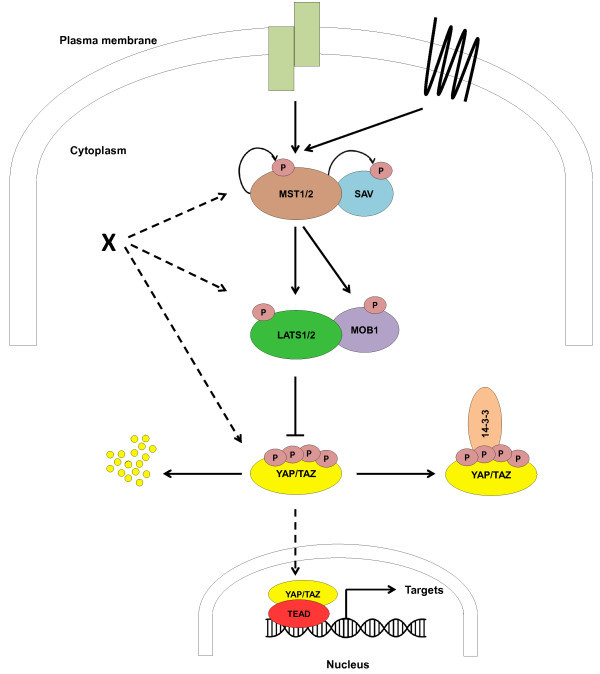
**The Hippo signalling core cassette in mammals.** In response to upstream signals (coming from GPCRs and other plasma membrane associated factors), MST1/2 are activated by phosphorylation. Phosphorylated MST1/2 in complex with the scaffolding protein SAV then activates LATS1/2 kinases by phosphorylation. Activated LATS1/2, associated with their co-activator MOB1, hyperphosphorylate YAP/TAZ on different sites. These YAP/TAZ phosphorylation events create a 14-3-3 binding site that causes the cytoplasmic retention of YAP/TAZ (mediated by Ser127 phosphorylation of YAP) and a separate phospho-degron that mediates the proteasomal degradation of YAP/TAZ (mediated by Ser381 phosphorylation of YAP). When the Hippo pathway is inactive, YAP/TAZ are not phosphorylated by LATS1/2 allowing the transcriptional co-activators YAP/TAZ to accumulate in the nucleus which can result in the transcription of specific target genes involved in cell cycle, apoptosis and differentiation control. Of note, the MST1/2-LATS1/2-YAP/TAZ axis can also be influenced by additional factors (depicted as X) on each individual signalling level.

The main function of the Hippo pathway is to regulate in a negative fashion the transcriptional co-activators Yes associated protein (YAP) and transcriptional co-activator with PDZ-binding motif (TAZ; also known as WWTR1) [3,4]. The core of the Hippo pathway consists of the mammalian Ste20-like serine/threonine kinases 1/2 (MST1/2), members of the Ste20 group of protein kinases [5], the large tumour suppressor 1/2 serine/threonine protein kinases (LATS1/2), members of the AGC kinase family [6,7], as well as their adaptor proteins Salvador (SAV; also termed WW45) [8] and Mps-one binder 1 (MOB1) [9]. Mechanistically, activated MST1/2 kinases associate with their scaffolding partner SAV and phosphorylate LATS1/2 and MOB1, resulting in increased LATS/MOB1 complex formation and LATS1/2 activation (Figure 1). Activated LATS1/2 kinases then phosphorylate YAP/TAZ on different sites, leading to the inactivation of YAP/TAZ by cytoplasmic sequestering and/or proteasome-mediated degradation (Figure 1). In case the MST1/2-SAV-MOB1-LATS1/2 signalling axis is inactive, YAP/TAZ can accumulate in the nucleus and function as transcriptional co-activators by interacting with transcription factors such as the TEA domain family members (TEADs; also known as TEFs) [1,4]. Key downstream targets of YAP/TAZ are regulators of cell cycle, apoptosis, and differentiation, although currently the precise transcriptional programmes of YAP/TAZ are not fully defined [4]. It is noteworthy that YAP/TAZ also interact with SMADs and other transcription factors in the context of the crosstalk between Hippo signalling and pathways such as Wnt and TGFβ signalling. Due to the emphasis of this manuscript, we refer the reader to other reviews to obtain an overview of these topics [10,11]. Furthermore, we would like to stress that different Hippo branches can function upstream of YAP/TAZ [1,3,12], but we focus here on discussing the main MST1/2-SAV-MOB1-LATS1/2-YAP/TAZ axis (Figure 1).

## Review

### Hippo signalling in cancer

The current view in the Hippo signalling field is that factors contributing to the inactivation of the proto-oncogenic YAP/TAZ proteins most likely represent tumour suppressor genes (TSGs), whereas activators/facilitators of YAP/TAZ functions are very likely to be proto-oncogenes. Given that these TSG vs. oncogene concepts have recently been summarised by excellent reviews on Hippo signalling in cancer [3,12], we will discuss in this subsection only some selected cancer-related points before highlighting non-cancer related pathologies upon deregulation of Hippo signalling.

In full support of TSG functions for the core components MST1/2, SAV and MOB1, loss of MST1/2, SAV, or MOB1 in mice results in the development of different tumour types, while YAP overexpression is sufficient to cause tumour formation (summarised in [3]). The development of tumours in LATS1 null mice has been reported more than 15 years ago, but this specific research aspect has not been pursued further since then. To our knowledge, conditional LATS1, LATS2 or LATS1/2 null mice have not been reported with respect to tumour development. Nevertheless, studies using mammalian cell lines support a role of LATS1/2 as TSGs (summarised in [6,13]). Unfortunately, the lack of LATS1/2 animal studies has hindered the definition of how far the MST1/2-SAV-MOB1-LATS1/2-YAP axis is responsible for tumour formation in transgenic MST1/2, SAV, MOB1, or YAP mice. Current evidence actually suggests that this axis does not always play a central role in animal models. For example, MOB1-deficient animals [14] develop the broadest range of tumours amongst all mice carrying manipulations of Hippo signalling components, suggesting that factors other than MST1/2, SAV, or LATS1/2 might play additional key roles [3,15]. As another example, liver specific ablation of MST1/2 causes liver tumours by YAP deregulation without any apparent role of LATS1/2 [16], while thymocyte specific deletion of MST1/2 results in thymic egress through a mechanisms not involving LATS1/2-YAP signalling [17]. These two studies strongly suggest that factors other than LATS1/2 function downstream of MST1/2 signalling. Whatever the case, it is undisputed that the deregulation of mammalian Hippo signalling components is implicated in tumour formation in spite of these findings [3].

Although Hippo signalling activities are clearly altered in human cancers, only few germline and somatic mutations of Hippo signalling components have been described so far, with the exception of YAP/TAZ amplification [3,4,12]. Considering recently reported genome wide screens for human cancer genes [18-20], none of the Hippo core components would have been defined as major TSGs or proto-oncogenes. Unlike well-defined oncogenic (e.g. c-kit) or tumour suppressor pathways (e.g. p53), no human cancers have been attributed to mutations or loss of the core signalling components of the Hippo pathway [3]. Given the observed redundancies for MST1/2, LATS1/2 and MOB1 (MOB1 refers to MOB1A and MOB1B, two independent genes in the genome [9]), it is unlikely that homozygous loss of MST1 and MST2 (or LATS1/2 or MOB1) can occur, since a total of four gene copies would have to be lost per signalling factor (e.g. both copies of MST1 and MST2). In support of this notion, biallelic loss of MST1 (also known as STK4) is not sufficient to cause human malignancies [21]. Given these puzzling findings in human samples, the following key questions with respect to human Hippo signalling remain unanswered: To what extent and how frequent is Hippo signalling deregulated in human tumours? How is Hippo signalling most frequently deregulated? Which cancer subtypes are mostly affected (or even caused) by deregulated Hippo signalling?

There are different reasons that may help to address these questions and also explain the lack of direct mutations in Hippo components. The first explanation could be the deregulated crosstalk of Hippo signalling with oncogenic pathways, such as WNT or mTOR [3]. A second possibility is that Hippo signalling might be affected by cumulative haploinsufficiency combined with triplosensitivity [18], although this type of analysis would have to be expanded to take redundancies into account. Third, perhaps the main defects in the Hippo signalling core can be attributed to altered post-translational modifications (PTMs) at the protein level, in which case genomic data cannot be used. In this context, we believe that it is time that regulatory phosphorylations of MST1/2, LATS1/2, MOB1, and YAP/TAZ [6] are to be carefully examined in the clinic. In addition, novel regulatory PTMs of YAP [22-24] should be included in a clinical setting to define the role of methylation and acetylation in the regulation of YAP/TAZ. Future clinical research into YAP/TAZ regulation may also need to consider circadian cycles, since the SCF β-TRCP E3 ligase promoting YAP/TAZ degradation [25,26] is known to play a role in circadian rhythms [27].

Another point worth mentioning is that YAP does not always function as a proto-oncoprotein [28,29]. Current evidence suggests that YAP performs oncogenic or tumour suppressive functions dependent on the breast cancer subtype [28,30]. This is not only valid for human breast cancer but has also been observed in colon cancer. Camargo and colleagues described a growth suppressive function of YAP in the mouse intestine and a silencing of YAP in a subset of human colorectal carcinomas [31,32]. In contrast, other studies observed an upregulation of YAP in samples of human colon cancers [4], as well as the need for YAP in β-catenin driven human colon cancer cell line survival and transformation [33]. Therefore, future studies should aim to define the tumour suppressive and/or proto-oncogenic functions of YAP (and possibly also TAZ) based on cancer subtype profiling. Most likely, Hippo signalling dependent- and independent mechanisms of YAP/TAZ regulation involving mechanical and cytoskeletal changes [34-38] will also need to be examined to fully understand the clinical situation.

Finally, in the context of Hippo signalling and cancer, the recent progress on deciphering the Hippo pathway protein-protein interactome should be mentioned [39]. Five independent studies systematically examined protein-protein interactions (PPIs) within the conserved Hippo-YAP/TAZ pathway [40-44]. These studies overwhelmingly illustrate that Hippo signalling represents a signalling network rather than a clear cut signal transduction cascade. For example, the scaffolding factors RASSF1-4 interact with MST1/2 [43], while RASSF8 is associated with YAP/TAZ [40,43]. Furthermore, two additional Ste20-like kinases, namely MST3 and MAP4K4, have been linked to the Hippo interactome in addition to MST1/2 [39], illustrating that different RASSF proteins and Ste20-like kinases will need to be considered in future studies. However, the endpoint of these multiple and diverse PPIs has remained the regulation of YAP/TAZ. Therefore, these studies have provided novel insight into putative functional modules (e.g. the NEK4, PLK1 and/or Citron kinases) that could be exploited for novel therapeutic approaches to manipulate YAP/TAZ activities, in addition to establishing a vast playground for mechanistic studies of Hippo signalling upstream of YAP/TAZ.

### Hippo signalling in non-cancer pathologies

While the role of Hippo signalling in tumour development is gaining more and more attention [3,12], non-cancer abnormalities involving Hippo components have only been studied to a limited extent. Similar to studies of Hippo signalling in cancer [3], our current understanding of Hippo signalling in non-cancer pathologies is mainly based on animal studies (summarised in Tables 1 and 2). Before discussing these disease links in more detail, we would like to stress two points. First, since MST1/2 and YAP/TAZ models have been studied the most exhaustively, we will focus on mainly reviewing these two signalling hubs. Second, we wish to bring to the reader’s attention that mice and humans tend to develop a different range of disease subtypes [3], hence one has to be careful when extrapolating information about Hippo signalling from animal models to human diseases. In this context, we would like to draw the reader’s attention to the fact that actual non-cancer human pathologies involving Hippo core components have only been described in the immune system, Alzheimer's disease, and glaucoma, while all other conditions described below are a result of experimentally induced disease in animals (Tables 1 and 2), awaiting confirmation of their existence in human diseases.

**Table 1:** Summary of non-cancer mammalian pathologies in which MST1/2 kinases are involved

**Tissue**	**Protein**	**Model**	**Pathology**	**References**
Liver	MST1/2	MST1/2 mutant conditional mouse	Hepatomegaly	[16,45,46]
Heart	MST1	Mouse model of Arrhythmogenic Cardiomyopathy and human samples	Arrhythmogenic Cardiomyopathy	[47]
		Neonatal rat ventricular myocytes	Heart failure, ischemic heart disease, dilated cardiomyopathy and cardiomyocyte apoptosis	[48-50]
		Dominant negative MST1 mice and MST1 -/- mice	Cardiac dysfunction	[51]
Muscle	MST1	MST1 deficient mice	Neurogenic muscle atrophy	[52]
Brain	MST1	Mouse model of Amyotrophic Lateral Sclerosis	Amyotrophic lateral sclerosis (ALS)	[53]
Pancreas	MST1/2	MST1/2 mutant conditional mouse	Reduction of pancreatic mass, exocrine pancreas disorganization and pancreatitis-like autodigestion	[54,55]
Thymus	MST1	MST1 deficient patients or with homozygous mutations	Immunodeficiency, T and B cells lymphopenia	[21,56]
		MST1 deficient mice	Defective lymphocyte trafficking and thymocyte egress. Autoimmune-like disorders. Impaired development and function of regulatory T cells. Low numbers of mature naïve T cells.	[57-60]
	MST1/2	MST1 and MST2 deficient mice	Autoimmune disease (skin lesions around the eyes, lymphocytes infiltration, colitis)	[17,61]
Lung	MST1/2	MST1/2 mutant conditional mouse	Respiratory distress syndrome	[62]
	MST1	Rat model of Hypoxic pulmonary vascular remodelling	Pulmonary arterial hypertension	[63]

**Table 2:** Summary of non-cancer mammalian pathologies in which YAP/TAZ proteins are involved

**Tissue**	**Protein**	**Model**	**Pathology**	**References**
Liver	YAP	Mouse models of inducible active YAP1 in the liver	Increase in liver size	[64,65]
Heart	YAP/TAZ	SCA-1-/- human cardiac progenitor cell line	Infarct	[66]
		Cardiac-specific YAP or TAZ knockout mice. Mouse model of inducible active YAP1 in the heart	Loss of function results in impaired neonatal heart regeneration and lethal cardiomyopathy. Activated YAP enhances cardiac regeneration and improves function of ischemic hearts	[67]
	YAP	Mouse models of arrhythmogenic cardiomyopathy and human samples	Arrhythmogenic Cardiomyopathy	[47]
		Mouse models of cardiomyocyte-specific homozygous inactivation of YAP in the postnatal heart	Increased myocyte apoptosis and fibrosis, dilated cardiomyopathy, and premature death.	[68]
Muscle	YAP	Mouse models of inducible active YAP in the skeletal muscle cells	Loss of body weight, gait impairments and kyphosis. Skeletal muscle atrophy.	[69]
Brain	YAP/TAZ	Rat model of chronic constriction sciatic nerve injury	Neuropathic pain	[70]
	YAP/TAZ	Mammalian cell lines	Alzheimer’s disease	[71]
Pancreas	YAP	MST1/2 mutant conditional mouse	Reduction of pancreatic mass, exocrine pancreas disorganization and pancreatitis-like autodigestion	[54,55]
		Mouse models of inducible active YAP1 in the pancreas	Pancreas increased in total size and acinar cells showed penetrant ductal metaplasia	[64]
Skin	YAP/TAZ	Mice model of wound healing	Wound healing	[72]
	YAP	Mouse models of inducible active YAP1 in the skin	Thickening of the epidermis and increased number of proliferating cells	[64]
Eye	YAP/TAZ	Primary human trabecular meshwork cells	Glaucoma	[73]
Ovary	YAP/TAZ	Mouse model of ovarian fragmentation, ovarian explant and follicle cultures. Primary ovarian insufficiency patients	Primary ovarian insufficiency and polycystic ovarian syndrome	[74]

Human patients with MST1 deficiency have an impaired immune system, hence suffering from immunodeficiency and lymphopenia [21,56]. Accordingly, MST1 deficient animals display a range of lymphocyte associated defects, ranging from defective lymphocyte trafficking to impaired development and function of regulatory T cells (summarised in Table 1). MST1 and MST1/2 conditional knock-out animals consequently exhibit features of autoimmune disease. Cumulatively, these reports strongly support the notion that MST1/2 kinases are important players in the mammalian immune system, while YAP/TAZ have not been associated with any thymus/immune system related roles (compare Tables 1 and 2).

Using mouse genetics, MST1/2 and YAP/TAZ have also been implicated in pathologies of the brain (Tables 1 and 2). MST1 deletion in an amyotrophic lateral sclerosis (ALS) mouse model delayed disease onset and prolonged survival of mice, thereby linking MST1 to neurodegeneration in ALS [53]. YAP/TAZ nuclear accumulation (which can be indicative of increased YAP/TAZ activities) was elevated upon peripheral nerve injury in a chronic nerve injury animal model [70]. It was further reported that YAP/TAZ function together with the amyloid-beta protein precursor, which is implicated in Alzheimer's disease (AD) [71]. Specifically, amyloid-beta protein precursor activates gene transcription through Mint3-TAZ and Mint3-YAP interactions [71]. These findings suggest that Hippo signalling might also play a role in ALS, neuropathic pain and AD.

Altered MST1 and YAP/TAZ activities have also been associated with heart defects (Tables 1 and 2). Moreover, mice with heart specific deletion of MST1/2 or YAP during embryonic development display defective heart development [75-77]. LATS1/2 or SAV loss also affect heart development [75,78], supporting the notion that Hippo signalling is required for normal heart formation. YAP overexpression results in increased proliferation of cardiomyocytes [67,68,76,77]. SAV deletion in the adult mouse heart also causes increased cardiomyocyte proliferation with elevated YAP expression [79], suggesting that cardiomyocyte proliferation is under the tight control of Hippo signalling. Furthermore, these results indicate that deregulating Hippo signalling might be beneficial for heart regeneration upon injury. In support of this, mice with heart specific YAP depletion were defective in heart regeneration [67], while LATS1/2 or SAV conditional knock-out animals displayed increased regenerative capacities [79]. In summary, these studies strongly indicate that the status of mammalian Hippo signalling modulates the potential of myocardial regeneration after injury.

Intriguingly, YAP is not only a regulator of cardiomyocyte proliferation, but also plays a significant role in skeletal muscle. Overexpression of YAP interferes with the differentiation of myoblasts into myotubes *in vitro*[80] and prevents the differentiation of satellite cells (stem cells of skeletal muscle) and myoblasts *in vivo*[81]. Therefore, it was speculated that YAP overexpression might be sufficient to drive excessive skeletal muscle formation. However, prolonged YAP overexpression resulted in skeletal muscle degeneration resembling human centronuclear myopathy [69]. In support of this finding, MST1 deletion also results in muscle atrophy [52]. Thus, inactivation of Hippo signalling (hyperactivation of YAP) seems to have detrimental effects on skeletal muscle homeostasis by causing atrophy and muscle deterioration (Tables 1 and 2).

Loss of MST1/2 function and overexpression of YAP have additionally been linked to pancreatic abnormalities (Tables 1 and 2). YAP overexpression results in ductal metaplasia in the pancreas besides causing severe abnormalities in the colon, skin, and liver [64,65]. Unexpectedly, conditional deletion of MST1/2 in the pancreas did not cause the same phenotype, but rather phenocopied pancreatitis in mice [54,55]. However, this phenotype was still connected with the regulation of YAP by MST1/2 signalling, since loss of MST1/2 resulted in a smaller pancreas due to postnatal reactivation of YAP expression, triggering undesired postnatal de-differentiation of pancreatic cells [54,55]. In summary, these studies show that the MST1/2-YAP axis of mammalian Hippo signalling is required to maintain postnatal homeostasis in the pancreas.

In other tissues the picture is different, since MST1/2 and YAP appear to function independently of each other. In the epidermis, YAP, but not MST1/2, play roles in the skin (compare Tables 1 and 2). Camargo and colleagues initially observed that YAP1 overexpression is sufficient to cause severe abnormalities in the skin of mice [64], and later reported that YAP is essential for normal skin homeostasis by regulating the epidermal stem cell pool, while MST1/2 are dispensable for normal skin biology [82]. Moreover, YAP/TAZ nuclear accumulation was markedly increased upon wound healing of epidermal injury, and YAP/TAZ depletion was sufficient to impair the rate of wound closure [72]. These observations suggested that Hippo signalling is also important for skin wound healing, although the MST1/2-LATS1/2 axis does not seem to play a key role [82].

MST1/2 signalling further regulates normal lung functionality (Table 1). Mice specifically lacking MST1/2 in the respiratory epithelium exhibited phenotypes that are very reminiscent of peripheral lung immaturity and respiratory distress syndrome (RDS) which is the leading cause of mortality in preterm babies [62]. Another study found that microRNA miR-138 regulates MST1 expression, thereby linking MST1 to hypoxic pulmonary vascular remodelling in rats [63], thereby suggesting MST1 supports normal lung development/homeostasis in rodents. Importantly, YAP does not seem to play a major role in lung development [62] but rather has been reported to play a significant part in diseases affecting the eye and ovary (Table 2). On the one hand, YAP/TAZ might be relevant as mechanotransducers in patients suffering from glaucoma, an eye disease that damages the optic nerve which impairs vision and sometimes leads to blindness [73]. On the other hand, disruption of Hippo signaling (hyperactivation of YAP/TAZ) has been linked to increased success rates in infertility treatments [74], suggesting that transient overactivation of YAP/TAZ can increase fertility.

Taken together, components of the MST1/2-SAV-MOB1-LATS1/2-YAP/TAZ axis are required to prevent pathological conditions that are not related to tumour formation (Tables 1 and 2). In some tissues, such as skeletal muscle or the pancreas, loss of Hippo signalling (hyperactivation of YAP/TAZ) is detrimental to the affected tissue, while in other organs, such as the heart or brain, inhibition of Hippo signalling is beneficial for injury response and disease delay. Therefore, selective manipulation of the Hippo pathway could be suitable for targeted therapy approaches in selected patient populations.

### Hippo signalling as a therapeutic target

#### Hippo signalling as an anti-cancer target

As already mentioned, the core components of Hippo signalling are essentially unaffected by genetic aberrations [3], suggesting that reactivation of the Hippo pathway in cancer cells might restore the proper inhibition of YAP/TAZ by Hippo signalling. This reactivation might involve different routes [12,37,83], some of which we will summarise here.

In the context of the recently mapped Hippo PPI network [40-44], the identification of cancer-enabling PPIs as potential therapeutic targets could provide a platform for the development of novel anti-cancer drugs. While the development of PPI inhibitors is a challenging task, it is still a feasible option as inhibitors of cancer-enabling PPIs have already entered clinical trials [84]. However, when considering Hippo signalling upstream of YAP/TAZ, the Hippo community will first have to define whether loss or gain of specific PPIs can act as major drivers of cancer before PPIs upstream of YAP/TAZ can be exploited for the development of novel therapeutics.

Currently, the only pre-clinical lead compound targeting a cancer driving PPI in Hippo signalling comes from studies addressing the YAP/TAZ interaction with the TEAD transcription factors. Since YAP/TAZ are the key downstream effectors of mammalian Hippo signalling [3,4] and their oncogenic actions can depend on their PPI with TEADs [82,85-90], the Pan laboratory examined the role of the YAP-TEAD interaction in murine liver tumours [91]. Significantly, they found that expression of dominant negative TEAD2 prevents YAP-driven cancer [91] without causing severe liver abnormalities [92]. More importantly, Pan and colleagues showed that verteporfin (VP), a FDA approved photosensitizer in the treatment of macular degeneration, interferes with formation of the YAP/TEAD complex, blocking YAP-driven liver overgrowth [91]. Recently, a naturally occurring antagonist of YAP-TEAD complex formation has provided a further lead for potential pharmacological intervention with YAP/TAZ activities [93,94]. The Tondu domains of vestigial-like family member 4 (VGLL4) interact directly with YAP, thereby preventing YAP-TEAD interactions [93,94], and a VGLL4-mimicking peptide disrupting YAP-TEAD interaction suppressed tumour growth in mice [93]. Cumulatively, these studies indicate that pharmacological intervention with YAP/TAZ-TEAD complex formation could be a feasible therapeutic approach with limited side effects. Co-crystal structures of YAP-TEAD are available [12], enabling the rationale design of small molecule inhibitors and the integration of findings indicating YAP and TAZ interact through different residues with TEAD [95]. Given that in mouse models reduced YAP activity negatively interferes with tumour growth [12] and that YAP depletion reduces the metastatic potential of human breast cancer cells [86,96], the development of an antagonist of the YAP-TEAD interaction could have significant therapeutic potential in the treatment of YAP/TAZ-driven cancers. Furthermore, since increased YAP/TAZ activities can trigger epithelial-mesenchymal transition (EMT) [97-101], a YAP/TAZ antagonist might influence the cellular plasticity in carcinomas, thereby decreasing therapeutic resistance, tumour recurrence and metastatic progression [102,103]. However, in this context one should note that the anti-tumour activity of compounds, such as VP or VGLL4 peptides, is yet to be examined in the setting of established tumours.

Another approach for the reactivation of Hippo signalling could be by increasing the activities of MST1/2 and/or LATS1/2 kinases functioning upstream of YAP/TAZ. Since MST1/2 and LATS1/2 kinases are regulated by multiple PPIs, which directly or indirectly affect their kinase activities [1,6,8,9,104], an in-depth characterisation of their main regulatory PPIs should provide valuable information for the development of novel drugs targeting the Hippo-YAP/TAZ pathway [83]. For example, by stimulating the activating PPI between MOB1 and LATS1/2 [9], an efficient decrease of YAP/TAZ activities might be obtained. We envision that this could be achieved by either increasing MOB1 phosphorylation by MST1/2, known to increase LATS1/2-MOB1 interactions [105], or by generating a MOB1-independent active LATS variant. In this sense, modified LATS kinases functioning independent of MOB1 and MST1/2 signalling maybe can be designed as recently described for the LATS-related NDR1 kinase [106]. Subsequently, using CRISPR-Cas9-mediated genome editing these kinase versions could be introduced into selected cancer tissues, similar to the recently reported restoration of Fah wild-type function to repair liver disease [107]. Nevertheless, the transient treatment with selective modulators of PPIs will most likely represent the safer option over permanent genome editing in this LATS1/2-MOB1 setting.

Alternatively, one should consider pharmacological inhibition of inhibitors of MST1/2 and/or LATS1/2 kinases functioning upstream of YAP/TAZ. Intriguingly, Dedhar and colleagues recently reported that integrin-linked kinase (ILK) plays a role in suppressing the Hippo pathway [108]. More specifically, they showed that ILK inhibition in human tumour cells results in MST1 and LATS1 activation with concomitant inactivation of YAP/TAZ activities. Even more importantly, Serrano et al. provided evidence indicating that pharmacological inhibition of ILK suppresses YAP activation and tumour growth in an animal model [108]. Thus, ILK is an attractive target for cancer therapy in patients with intact Hippo signalling.

As another alternative to the reactivation of Hippo signalling approach, one could consider stimulators of YAP/TAZ activities as drug targets. Homeodomain interacting protein kinases (HIPKs) [109] and salt-inducible kinases (SIKs) [110] represent possible drug targets to blunt YAP (and possibly also TAZ) activity, as both kinases promote YAP activity in human cells. However, the molecular mechanism(s) of how these kinases promote YAP activity in human cells are to be understood in detail before rational drug design approaches can be initiated. Another alternative to restrain YAP/TAZ activities could be based on the recently established link between the Hippo-YAP/TAZ pathway and G-protein coupled receptor (GPCR) signalling [111-114]. Since many currently used therapeutic compounds target GPCR signalling directly or indirectly [115,116], GPCR-Hippo signalling represents an attractive druggable target [83]. However, the suitability of GPCR agonists and antagonists for clinical applications in YAP/TAZ-driven human cancers has yet to be determined.

Intriguingly, YAP/TAZ regulation involves more than the Hippo core kinases, MST1/2 and LATS1/2, which has the potential to open novel routes for therapeutic intervention [37]. For example, a recent report showed that YAP/TAZ activities are controlled by the mevalonate pathway [117], suggesting that FDA-approved cholesterol biosynthesis inhibitors, such as Statins, have the potential to target YAP/TAZ in malignant cancer cells. The FDA-approved broad-acting tyrosine kinase inhibitor dasatinib might also be used to treat β-catenin/YAP-driven colon cancer cells [33]. Moreover, YAP expression levels affect the response to tamoxifen in specific breast cancer subtypes [30]. YAP depletion further sensitizes human cancer cells to anti-cancer agents, such as cisplatin or the EGFR tyrosine kinase inhibitor erlotinib [118], and increased YAP/TAZ levels correlate with taxol and cisplatin resistance [100,119,120]. Therefore, pharmacological inhibition of YAP/TAZ might be achieved through already available FDA-approved clinical compounds or combinations therewith.

#### Hippo signalling as a target in non-cancer settings

While the inhibition of YAP/TAZ activities is desirable for cancer treatments, the opposite is considered true for heart regeneration, where YAP is needed for neonatal heart regeneration in mice [67,79] and YAP overexpression promotes heart regeneration after myocardial injury [67]. Therefore, pharmacologically elevated YAP activity could accelerate tissue repair following injuries such as myocardial infarcts [12]. Since MST1 overexpression in the heart resulted in cardiac dysfunction [121] and overexpression of dominant-negative MST1 or LATS2 improved cardiac function after injury [122,123], the elevation of YAP activity could be achieved by transiently inhibiting MST1/2 and/or LATS1/2 kinases through direct kinase inhibition or by interfering with activating PPIs such as LATS1/2-MOB1 interactions. Taken together, MST1/2 and LATS1/2 kinases should be considered as potential drug targets for regenerative medicine, applied transiently in conditions such as recovery from myocardial injury.

#### Challenges for future therapeutic approaches

Pharmacological inhibition of MST1/2 and/or LATS1/2 is of interest in the clinical setting of recovery from myocardial injury as transient YAP activation in cardiomyocytes could expand the cardiomyocyte cell pool during therapeutic heart regeneration [12]. In this context, transiently amplified YAP/TAZ activities might also help to mobilise and increase stem and progenitor cell populations and maybe even be beneficial for the re-programming of differentiated human cells. However, while increased YAP/TAZ activities are desirable for tissue regeneration, the effects of prolonged YAP/TAZ hyperactivation should not be underestimated. On the one hand, increased YAP activity can result in severe abnormalities of the liver, colon, skin, and pancreas [64,65]. On the other hand, constitutively active YAP can result in muscle atrophy and deterioration [69]. In general, prolonged elevation of YAP/TAZ activities has the potential to trigger uncontrolled expansion of stem cell pools, cellular transformation of epithelial cells, and undesired dedifferentiation of functional units such as muscle fibres.

Along this line, prolonged decrease of YAP/TAZ activities is most likely detrimental to normal stem cell pools in patients. While we currently do not understand the long term consequences of sustained YAP/TAZ inhibition, the central role of YAP/TAZ in mammalian stem cells is undeniable [31]. YAP and TAZ are critical regulators of stem cell pluripotency in murine [124] and human cells [125,126]. Thus, although YAP/TAZ depletion has the potential to inhibit cancer stem cell expansion in a clinical setting [97], it is very likely that YAP/TAZ inhibition would also negatively affect essential stem cell pools in non-cancerous tissues. In summary, increased YAP/TAZ activities are associated with stem cell expansion that is coupled with inhibition of differentiation, while reduction of YAP/TAZ activities results in the opposite effect. Therefore, all clinical approaches aiming to manipulate Hippo-YAP/TAZ signalling activities will have to be finely balanced in order to effectively manage undesirable long term side effects.

## Conclusions

Members of the Hippo pathway are emerging targets in anti-cancer treatments and regenerative medicine. In particular, interference with YAP/TAZ-TEAD interactions is of central interest in the development of novel anti-cancer agents. In this context, already FDA-approved drugs might serve as tool compounds to develop selective inhibitors blocking YAP/TAZ-TEAD interactions. Further FDA-approved agents, initially designed to target enzymatic activities in GPCR signalling or the mevalonate pathway, have also the potential to interfere with YAP/TAZ activities indirectly. Since the Hippo pathway is regulated by many PPIs which potentially could serve as targets for intervention, selective PPIs might also be used to design pharmacological modulators of Hippo signalling. Therefore, the recent dissection of the Hippo pathway protein-protein interactome could be instrumental for the discovery of novel therapeutic approaches to manipulate YAP/TAZ activities directly or indirectly. Potentially by deciphering the key regulatory and disease-relevant PPIs functioning upstream of YAP/TAZ, future studies will provide novel insights into functional modules that might be exploitable for novel therapeutic approaches to manipulate YAP/TAZ activities.

Based on the organ and cell-type specific benefits or detrimental consequences of diminished Hippo signalling (Tables 1 and 2), tissue specific and whole organism side effects always need to be considered in any upcoming clinical application. For example, transient inhibition of MST1 kinase activity is desirable when recovering from myocardial injury, while prolonged MST1 deficiency is detrimental to the human immune system. In general, any manipulation of the Hippo-YAP-TAZ pathway will have to be addressed very cautiously to ensure that the stem cell and progenitor pools in vital organs and tissues of patients are not significantly altered upon drug treatment. Nevertheless, considering the very promising progress in our understanding of Hippo signalling with respect to many human-associated diseases, such as cancer, hearts defects, brain-related pathologies, and immune deficiencies, we are confident that intensive research efforts over the coming years will reveal the full potential of manipulations of the Hippo pathway in the prevention and treatment of a broad range of human diseases.

## Abbreviations

YAP: Yes associated protein; TAZ: Transcriptional co-activator with PDZ-binding motif; MST1/2: Mammalian Ste20-like serine/threonine kinases 1/2; LATS1/2: Large tumour suppressor 1/2; SAV: Salvador; MOB1: Mps-one binder 1; TEAD: TEA domain family member; TSG: Tumour suppressor gene; PTM: Post-translational modification; PPI: Protein-protein interaction; ALS: Amyotrophic lateral sclerosis; AD: Alzheimer's disease; RDS: Respiratory distress syndrome; VP: Verteporfin; VGLL4: Vestigial-like family member 4; EMT: Epithelial-mesenchymal transition; HIPK: Homeodomain interacting protein kinase; SIK: Salt-inducible kinase; GPCR: G-protein coupled receptor.

## Competing interest

The authors declare that they have no commercial or other competing interests to disclose.

## Authors’ contributions

MG, VG, and AH. researched the literature and wrote the manuscript together. MG researched and designed the tables. VG created Figure 1. All authors read and approved the final manuscript.
